# Estimation and prediction on the economic burden of schistosomiasis in 25 endemic countries

**DOI:** 10.1186/s40249-025-01330-8

**Published:** 2025-06-16

**Authors:** Xian-Fa Chen, Qin Li, Robert Bergquist, Jin-Xin Zheng, Su-Ying Guo, Qiu-Feng Lan, Zheng-Ze He, Li-Juan Zhang, Chun-Li Cao, Jing Xu, Xiao-Nong Zhou

**Affiliations:** 1 National Institute of Parasitic Diseases at Chinese Center for Disease Control and Prevention (Chinese Center for Tropical Diseases Research); National Key Laboratory of Intelligent Tracking and Forecasting for Infectious Diseases; Key Laboratory On Parasite and Vector Biology, National Health Commission; WHO Centre for Tropical Diseases; National Center for International Research on Tropical Diseases, Ministry of Science and Technology, Shanghai, 200025 China; 2 School of Global Health, Chinese Center for Tropical Diseases Research, Shanghai Jiao Tong University School of Medicine, Shanghai, 200025 China; 3 Geospatial Health, Ingerod, Brastad, Sweden; 4 Hainan Center for Tropical Diseases Research (Hainan Sub-Center, Chinese Center for Tropical Diseases Research), Haikou, 570100 China

**Keywords:** Schistosomiasis, Elimination, Economic burden, Global burden, Macroeconomic model, Endemic country

## Abstract

**Background:**

Schistosomiasis is a neglected tropical disease, primarily prevalent in tropical and subtropical regions. It imposes a significant health and economic burden in low- and middle-income countries, but a study of its comprehensive economic impact of the disease at the global level has not been carried out. As this is essential for evidence-based decision-making, this study aims to estimate the macroeconomic burden of schistosomiasis in 25 endemic countries.

**Methods:**

We used a health-augmented macroeconomic (HAM) model, as well as observed data from 2010 to 2021 and projected data from 2022 to 2050, to model gross domestic product (GDP) under two scenarios: with and without schistosomiasis. The data were obtained from the Global Burden of Disease Study 2021 (GBD 2021), the World Bank database, the International Monetary Fund (IMF) database, the International Labour Organization (ILO) database, the United Nations Population Division's World Population Prospects 2022 database, the Barro-Lee Educational Attainment dataset, the Penn World Table (PWT) database, and relevant literature. The economic burden was quantified as the difference in GDP between these two scenarios. The HAM model considered: (i) the impact of schistosomiasis mortality and morbidity on labor supply; (ii) age and gender differences in education and work experience among schistosomiasis patients; and (iii) the impact of schistosomiasis treatment costs on physical capital accumulation. To be able to compare the purchasing power of different countries, we used international dollars (INT$), a hypothetical currency unit based on purchasing power parity.

**Results:**

We estimated the macroeconomic burden of schistosomiasis in 25 schistosomiasis endemic countries was INT$ 49,504 million [uncertainty interval (UI): 48,668–50,339] for the study period, using a 3% discount rate in the main analysis. The result implies that the economic burden of schistosomiasis across these 25 countries during study period is equivalent to 0.0174% (UI: 0.0171–0.0177) of total GDP. Among all schistosomiasis-endemic countries included, Egypt had the largest absolute economic burden (INT$ 11,400 million, UI: 11,221–11,578), followed by Brazil (INT$ 9779 million, UI: 9717–9841) and South Africa (INT$ 6744 million, UI: 6676–6811).

**Conclusions:**

The global economic burden of schistosomiasis remains substantial and is inequitably distributed among countries and regions. Our study highlights the need for increased investment and global collaborative efforts to control schistosomiasis and its associated health and economic burdens. By advancing the elimination of schistosomiasis, substantial economic returns can be achieved.

**Graphical Abstract:**

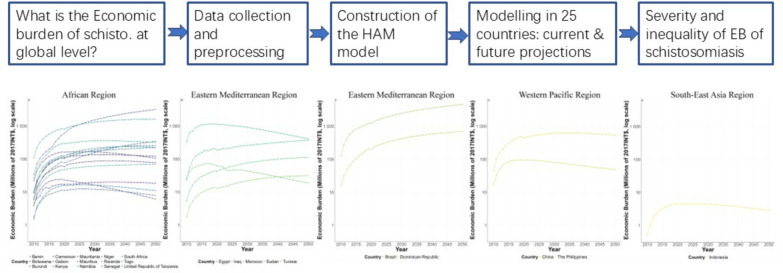

**Supplementary Information:**

The online version contains supplementary material available at 10.1186/s40249-025-01330-8.

## Background

Schistosomiasis, listed as a neglected tropical disease (NTD) by the World Health Organization (WHO), is a poverty-related parasitic disease, mainly affecting low- and middle-income countries (LMICs) in tropical and subtropical regions [1]. According to the Global Burden of Disease Study 2021 (GBD 2021) [2], the disease caused 1,746,333 disability-adjusted life years (DALYs) globally in 2021, resulting in 12,857 deaths. Schistosomiasis is mainly prevalent in Asia, Africa, and Latin America, with over 90% of DALYs occurring in Africa, making it the region with the most severe disease burden [3]. Although the disease is a predominantly chronic, it has also a considerable mortality. By country, Nigeria experienced the highest number of schistosomiasis deaths globally (1411), followed by the Democratic Republic of the Congo (1356) in 2021. In order to further reduce the global burden of NTDs including schistosomiasis, WHO released an update NTD Roadmap 2021–2030 in 2020, aims at achieving the elimination of schistosomiasis as a public health problem in 78 endemic countries by 2030 [4]. Currently, all endemic countries are accelerating efforts towards this goal [5].

In addition to causing a severe health burden, schistosomiasis imposes a substantial economic burden through reduced productivity, unemployment, labour loss, and decreased capital investment [6]. First, premature death and disability prevent individuals from participating in market activities, reducing the labour supply. While illness reduces the ability to perform normal work or leads to early retirement, decreasing working hours. Second, affected populations often exhibit lower savings rates, investment, and physical capital accumulation because they spend more resources on medical expenses. Therefore, investments in schistosomiasis screening, diagnosis, and treatment can yield substantial health and economic benefits, particularly in LMICs, which have lower schistosomiasis survival rates compared to high-income countries [7]. Comprehensive schistosomiasis economic burden assessments, as well as projections and understanding the variations in economic burden across countries, are crucial as it enables policymakers to develop targeted policies for effectively reducing schistosomiasis-related mortality and morbidity. Furthermore, such estimate assists the allocation of limited health resources efficiently and helps to build health systems capable of effectively responding to schistosomiasis outbreaks.

Previous studies on the economic burden of schistosomiasis have estimated the burden in one or a few countries. Most used the cost-of-illness approach (aggregating direct and indirect costs) or the value-per-statistical-life approach (multiplying the number of cases and deaths by the amount individuals would pay to avoid the risk) [8, 9]. However, these methods fail to account for economic adjustment mechanisms and cannot effectively guide resource allocation for schistosomiasis control efficiently. These are common limitations in disease burden estimations and projections, since the methods applied are static, failing to account for population dynamics driven by changes in morbidity and mortality as well as dynamic changes in physical capital accumulation driven by schistosomiasis treatment costs. Recently, studies have used a health-augmented macroeconomic (HAM) model to estimate the economic burden of non-communicable diseases, road traffic injuries and risk factors, which addresses economic cost mechanisms to guide policymakers with respect to prevention and control [10–13]. The HAM model is an economic framework that integrates health factors into traditional macroeconomic analysis by considering the impact of health on labour supply, productivity, and human capital, which can estimate how diseases and health conditions affect economic growth and development. This model allows policymakers to assess the economic implications of health policies, disease outbreaks, and healthcare investments, providing insights into the broader economic costs and benefits of health.

To our knowledge, no study has comprehensively assessed the global economic burden of schistosomiasis. To fill this gap, this study used a theory-based HAM model to estimate the economic burden of schistosomiasis for 25 countries from 2010 to 2021 and then predict how the economic burden would develop until 2050.

## Methods

We used HAM model, which measures the cost of disease through two scenarios of gross domestic product (GDP), to estimate the economic burden of schistosomiasis across 25 countries during 2010–2021, and to predict these burdens from 2022 to 2050 [14].

### Schistosomiasis endemic countries

A total of 25 countries endemic for one or more of the main schistosome species were selected from the 78 schistosomiasis endemic countries globally, to compare the economic burdens due to this infection (Table S1 of the Appendix 2). The criteria used for selection were as follows: (i) being a schistosomiasis endemic country; (ii) availability of reliable epidemiological data; (iii) all required variables for establishment of the HAM model during the study period (some missing data were interpolated); (iv) having a national schistosomiasis control programme, with elimination as the long-term goal.

### Data sources

This study adopted the disease definition and classification criteria for schistosomiasis from GBD 2021[15]. Data used in the study included epidemiological data, treatment cost data, economic data, population data, education data, and data on the labour force. Linear interpolation combined with nearest-neighbour interpolation was used to address missing data.

The epidemiological data included mortality, morbidity, years of life lost due to disability (YLD), and years of life lost (YLL) in each country and were sourced from the database of GBD 2021 by the Institute for Health Metrics and Evaluation of the University of Washington, Seattle, WA, USA. We estimated mortality, morbidity, and DALY of schistosomiasis in China adjusted based on a 1.5-fold discrepancy between the GBD 2021 estimates and China’s Schistosomiasis Surveillance System [16–18]. For the treatment cost, per-case treatment cost in a specific country proportional to per capita health expenditure, were estimated as suggested in previous studies [19]. The costs of schistosomiasis caused by *Schistosoma mansoni* and *S. japonicum* were collected separately, based on research results from Brazil and China [20]. Specifically, based on the per-case treatment cost of schistosomiasis in China, the proportion of treatment cost for schistosomiasis japonica to per capita health expenditure was estimated, and then extrapolated to calculate treatment costs in China, the Philippines and Indonesia. Additionally, using Brazil’s schistosomiasis mansoni treatment cost data, the treatment costs for other schistosomiasis-endemic countries prevalent with *S. mansoni* and *S. haematobium* were calculated.

Economic data included (i) GDP, health expenditure per capita, consumption as a percentage of GDP, and the saving rate obtained from the World Bank’s World Development Indicators database [21, 22]; (ii) physical capital stock and the elasticity sourced from the PWT database [23]. All economic estimates were converted to constant 2017 international dollars (INT$), which is a hypothetical currency unit used to compare the purchasing power of different countries based on purchasing power parity (PPP) rather than market exchange rates. This means adjustments for price differences between countries, making it more accurate for comparing economic output.

The population data for specific sex-age groups (5-year age groups) were obtained from the World Population Prospects 2022[24]. We assumed that the population aged 15–64 years was the working-age population [25]. The education data were obtained from the Barro-Lee Educational Attainment Database and the parameters for the Mincer equation were derived from Psacharopoulos and Patrinos for education level and Heckman for workforce experience [26–28]. Labour force participation rate data for specific sex-age groups were extracted from the International Labour Organization (ILO) [29].

### Model establishment

We developed the HAM model after all parameters were estimated (Table S2 of the Appendix 2), following previous studies of Bloom et al. and Chen et al. [14, 19] Generally, a HAM model framework to estimate the economic burden of schistosomiasis was established to quantify the impact of schistosomiasis on economic output by healthcare expenditures and productivity losses due to mortality and morbidity (Appendix 1). For each country, we conducted the following analysis:

Step 1: We identified the disease burden of schistosomiasis, based on mortality, morbidity, and treatment costs.

Step 2: We constructed economic output projections under two scenarios: a status quo scenario representing aggregate output under current conditions with no interventions to reduce schistosomiasis mortality and morbidity; and a counterfactual scenario representing aggregate output if schistosomiasis were completely eliminated at zero cost. Economic projections utilize a macroeconomic production function and can be decomposed into two components: projections of effective labour supply; and projections of physical capital accumulation.

Step 3: We cumulatively summed the difference in annual GDP between the two scenarios. The cumulative difference represents the total economic burden:

1 $$\Delta Y = \sum\nolimits_{t = 2010}^{2050} {\left( {\overline{Y}_{t} - Y_{t} } \right)}$$

### Production function

Considering the discrete time evolution (t = 1, 2, …) in the economy, building upon Lucas's work, we considered the following production function for each economy [30]:

2 $${Y}_{t}={A}_{t}{H}_{t}^{1-\alpha }{K}_{t}^{\alpha }$$

where ${Y}_{t}$ represents aggregate output; ${A}_{t}$ the level of technology in year t, which we assume evolves exogenously; ${K}_{t}$ the physical capital stock (e.g., machines, factory buildings, etc.); and ${H}_{t}$ total human capital. The parameter $\alpha$ is the elasticity of aggregate output with respect to physical capital. Solow's framework [on which the WHO's Economic Projections for Illness and Cost of treatment (EPIC) macroeconomic model was based] considers only physical capital and raw labour as factors of production. However, the aggregate production function recognizes that output is produced not only by these factors but also by effective labour, where health is a crucial determinant [31].

Physical capital evolves according to the following equation:

3 $${K}_{t+1}=\left(1-\delta \right){K}_{t}+{Y}_{t}-{C}_{t}-T{C}_{t}=\left(1-\delta \right){K}_{t}+{s}_{t}{Y}_{t}$$

where $\updelta$ represents the depreciation rate, ${S}_{t}$ the savings rate, ${TC}_{t}$ the treatment cost of schistosomiasis, and ${C}_{t}$ the quantity of consumption.

From Eq. 3, the savings rate can be defined as:

4 $${s}_{t}=1-\frac{{C}_{t}+T{C}_{t}}{{Y}_{t}}$$

where ${Y}_{t}$ represents total output used for three purposes: (i) paying for treatment costs, (ii) consumption, and (iii) savings.

We assumed that the working-age population is defined as those aged 15–64 years, and this population was further divided into ten 5-year age groups. Thus, we had 20 age-sex groups. Total human capital in the production function can be defined as the sum of the effective labour supply of each age-sex group:

5 $${H}_{t}={\sum }_{a}{h}_{t}^{a}{{l}_{t}^{a}N}_{t}^{a}$$

where ${N}_{t}^{a}$ represents the number of individuals in age group a, ${h}_{t}^{a}$ the average human capital of individuals in age group a, and ${l}_{t}^{a}$ the labour force participation rate in age group *a*.

Following the Mincer model, we constructed the average human capital for age group *a* based on education level and work experience:

6 $$\text{ln}{h}_{t}^{a}={\eta }_{1}y{s}_{t}^{a}+{\eta }_{2}\left(a-y{s}_{t}^{a}-5\right)+{\eta }_{3}{\left(a-y{s}_{t}^{a}-5\right)}^{2}$$

where ${\eta }_{1}$ is the semi-elasticity coefficient of human capital with respect to average years of schooling ${ys}_{t}^{a}$, and ${\eta }_{2}$, ${\eta }_{3}$ the semi-elasticity coefficients of human capital with respect to experience of the workforce $\left(a-y{s}_{t}^{a}-5\right)$ and experience of the workforce squared ${\left(a-y{s}_{t}^{a}-5\right)}^{2}$, respectively. Here, we assumed a school entry age 5 years throughout.

### The impact of schistosomiasis on labour supply

Referring to Bloom et al. and Chen et al., in the status quo scenario, labour supply can be obtained through ${L}_{t}^{a}={l}_{t}^{a}{N}_{t}^{a}$.

7 $${N}_{t}^{a}=\left[1-{\sigma }_{t-1}^{a-1}\right]{N}_{t-1}^{a-1}$$

In Eq. (5), ${\sigma }_{t}^{a}$ represents the overall mortality rate for age group* a* in year *t*. Mortality and morbidity reduce the effective labour supply. The reduction of population size ${N}_{t}^{a}$ reflects the impact of mortality. ${\sigma }_{d,t}^{a}$ represents the mortality rate in age group *a* due to schistosomiasis. ${\sigma }_{-d,t}^{a}$ represents the overall mortality rate from causes other than schistosomiasis. Then we have:

8 $$\left(1-{\sigma }_{t}^{a}\right)=\left(1-{\sigma }_{d,t}^{a}\right)\left(1-{\sigma }_{-d,t}^{a}\right)$$

In the counterfactual scenario, variables are represented with an overline. The population size for age group *a* in year *t* can be obtained using the following formula:

9 $${\overline{N}}_{t}^{a}=\left[1-{\sigma }_{-d,t-1}^{a-1}\right]{\overline{N}}_{t-1}^{a-1},$$

10 $${\overline{N}}_{0}^{a}={N}_{0}^{a},$$

11 $${\overline{N}}_{t}^{0}={N}_{t}^{0}$$

Referencing Bloom et al., the labour loss due to mortality accumulates year by year, as shown in the following formula:

12 $${\overline{N}}_{t}^{a}=\frac{{N}_{t}^{a}}{\prod_{\tau =0}^{\text{min}\left\{t,a\right\}-1}\left[1-{\sigma }_{d,t-1-\tau }^{a-1-\tau }\right]}$$

The reduction in labour force participation rate ${l}_{t}^{a}$ reflects the effects of morbidity, as individuals suffering from illness typically reduce their labour supply, either by reducing working hours or by leaving their jobs. Referencing Bloom et al., the labour force participation rate in the counterfactual scenario ${\overline{l}}_{t}^{a}$ can be calculated as:

13 $${\overline{l}}_{t}^{a}\simeq \frac{{l}_{t}^{a}}{\prod_{\tau =0}^{\text{min}\left\{t,a\right\}-1}\left[1-{p}^{\tau }{\sigma }_{d,t-1-\tau }^{a-1-\tau }{\xi }^{a-1-\tau }\right]}$$

where ${\xi }^{a}$ measures the magnitude of the morbidity effect relative to mortality. and $p$ the probability that a schistosomiasis patient cannot recover from the disease. So we used the efficacy of the WHO-recommended 40 mg/kg praziquantel treatment regimen to calculate this parameter value.

Because the impact of morbidity is difficult to estimate directly, we defined:

14 $${\xi }^{a}=\frac{loss\, of\, labor\, due\, to\, morbidity\, in\, age\, group\, a}{loss\, of\, labor\, due\, to\, mortality\, in\, age\, group\, a}$$

Next, we assumed that for each age group *a* in any given year, the following equation holds:

15 $${\xi }^{a}=\frac{YL{D}^{a}}{YL{L}^{a}}$$

where $YL{D}^{a}$ represents the years lived with schistosomiasis, and $YL{L}^{a}$ the years of life lost due to schistosomiasis. ${\xi }^{a}$ can be calculated using the corresponding DALYs data reported in the GBD2021.

### The impact of schistosomiasis on physical capital accumulation

Schistosomiasis also hinders physical capital accumulation, as savings are diverted to pay for part of treatment costs. According to Bloom et al. and Chen et al. [14, 19], physical capital accumulation in the counterfactual can be written as:

16 $${\overline{K} }_{t+1}={\overline{s} }_{t}{\overline{Y} }_{t}+\left(1-\delta \right){\overline{K} }_{t}$$

17 $${\overline{s} }_{t}{\overline{Y} }_{t}={s}_{t}{\overline{Y} }_{t}+\chi T{C}_{t}$$

By using this model, we first used data of the predicted GDP, effective labour supply, and physical capital stock in the status quo scenario (2010–2050) to calibrate the technological level parameter ${A}_{t}$. Then, the effective labour supply and physical capital stock in the counterfactual scenario were estimated, in combination with the estimated technological level, and finally calculated the counterfactual GDP.

### Pattern analysis on economic burden

*Spatiotemporal analysis:* To investigate the spatiotemporal patterns of the economic burden of schistosomiasis, both cross-sectional analysis and time-series analysis were performed. First, the cross-sectional analysis method was employed to compare the economic burdens of schistosomiasis across different countries or regions. Second, the time-series analysis method was applied to systematically assess the temporal trends of the economic burden, thereby elucidating its stage-specific characteristics [32].

*Sensitivity analysis:* We performed a sensitivity analysis by varying mortality and morbidity rates based on GBD 2021. The average mortality and morbidity rates were used as the baseline estimates. Best-case and worst-case estimates were calculated by applying the lower and upper bounds of the GBD mortality and morbidity data, respectively, which represent the 95% uncertainty interval (UI) of the GBD estimates [33]. Additionally, a sensitivity analysis was conducted by varying the discount rate. In the main analysis, a 3% discount rate was used, while additional analyses were performed using discount rates of 0%, 2%, 4%, and 5%[34]. Data analysis was performed using R (Version 4.3.0), with the development environment in RStudio (Version 2023.06). Data visualization was conducted using the ggplot2 package (Version 3.4.2).

## Results

### Economic burden of schistosomiasis from 2010 to 2021

We estimated the macroeconomic burden of schistosomiasis in 25 schistosomiasis-endemic countries from 2010 to 2021 to be INT$ 49,504 million (UI: 48,668–50,339), using a 3% discount rate in the main analysis (Table 1). The result implies that the economic burden of schistosomiasis across these 25 countries from 2010 to 2021 is equivalent to 0.0174% (UI: 0.0171–0.0177) of cumulative output, or the per capita economic burden of INT$ 1.673 (UI: 1.645–1.702). However, there were big variations at regional and country levels.

**Table 1:** Total economic burden, economic burden as a proportion of GDP, and per capita economic burden of schistosomiasis in 25 countries from 2010 to 2021, using a 3% discount rate

Region	Country	Economic loss, millions of 2017 INT$ (uncertainty interval)	Proportion of total GDP in 2010–2021, × 10^–3^ (uncertainty interval)	Per capita loss, 2017 INT$ (uncertainty interval)
AFRO	*Regional average*	1290 (604–2329)	1.881 (1.327–2.423)	9.652 (4.935–15.472)
	Benin	842 (823–861)	2.554 (2.496–2.611)	6.293 (6.151–6.435)
	Botswana	493 (454–533)	1.476 (1.357–1.596)	17.610 (16.187–19.032)
	Burundi	157 (154–160)	1.844 (1.812–1.876)	1.207 (1.186–1.227)
	Cameroon	1352 (1306–1398)	1.597 (1.542–1.651)	4.813 (4.649–4.977)
	Gabon	217 (173–262)	0.730 (0.580–0.880)	8.867 (7.042–10.692)
	Kenya	3535 (3511–3560)	1.748 (1.736–1.760)	6.223 (6.181–6.266)
	Mauritania	96 (87–105)	0.461 (0.418–0.505)	1.987 (1.801–2.174)
	Mauritius	576 (533–619)	2.201 (2.036–2.367)	37.135 (34.341–39.929)
	Namibia	852 (833–870)	3.645 (3.566–3.723)	30.764 (30.099–31.429)
	Niger	124 (118–131)	0.525 (0.498–0.553)	0.501 (0.475–0.527)
	Rwanda	983 (979–988)	4.411 (4.393–4.430)	6.927 (6.898–6.956)
	Senegal	874 (848–899)	1.898 (1.843–1.952)	4.982 (4.839–5.126)
	South Africa	6744 (6676–6811)	0.863 (0.854–0.872)	10.087 (9.986–10.188)
	Togo	370 (367–374)	2.530 (2.507–2.553)	4.070 (4.033–4.107)
	United Republic of Tanzania	2140 (2088–2191)	1.736 (1.694–1.777)	3.313 (3.233–3.393)
AMRO	*Regional average*	5612 (1446–9779)	0.598 (0.311–0.884)	7.739 (3.960–11.519)
	Brazil	9779 (9717–9841)	0.311 (0.309–0.313)	3.960 (3.935–3.985)
	Dominican Republic	1446 (1389–1502)	0.884 (0.849–0.919)	11.519 (11.067–11.971)
*EMRO*	*Regional average*	2862 (328–7209)	0.425 (0.164–0.748)	3.325 (0.962–6.711)
	Egypt	11,400 (11,221–11,578)	1.043 (1.026–1.059)	9.644 (9.493–9.795)
	Iraq	1734 (1658–1810)	0.482 (0.461–0.503)	3.819 (3.652–3.986)
	Morocco	352 (338–365)	0.129 (0.124–0.134)	0.841 (0.808–0.873)
	Sudan	714 (702–726)	0.383 (0.377–0.389)	1.519 (1.494–1.544)
	Tunisia	112 (106–119)	0.089 (0.084–0.094)	0.804 (0.758–0.851)
SEARO	*Regional average*	37 (30–44)	0.001 (0.001–0.002)	0.012 (0.01–0.014)
	Indonesia	37 (30–44)	0.001 (0.001–0.002)	0.012 (0.01–0.014)
WPRO	*Regional average*	2288 (889–3686)	0.242 (0.005–0.479)	1.501 (0.053–2.949)
	China	889 (887–891)	0.005 (0.005–0.005)	0.053 (0.053–0.053)
	The Philippines	3686 (3669–3703)	0.479 (0.476–0.481)	2.949 (2.936–2.963)
Total		49,504 (48,668–50,339)	0.174 (0.171–0.177)	1.673 (1.645–1.702)

AFRO: African Regional Office; AMRO: Regional Office for the Americas; EMRO: Eastern Mediterranean Regional Office; SEARO: South-East Asia Regional Office; WPRO: Western Pacific Regional Office

At the regional level, the American region was found to have the highest average of INT$ 5612 million (UI: 1446–9779) in terms of total economic burden. As a proportion of GDP, the African region had the highest average of 0.1811% (UI: 0.1327%–0.2423%). In terms of economic burden per capita, the African region had the highest average of INT$ 9.652 (UI: 4.935–15.472).

At the country level, Egypt had the largest absolute economic burden (INT$ 11,400 million, UI: 11,221–11,578), followed by Brazil (INT$ 9779 million, UI: 9717–9841) and South Africa (INT$ 6744 million, UI: 6676–6811). Considering the economic burden as a percentage of GDP, Rwanda (0.4441%, UI: 0.4393%–0.443%), Namibia (0.3645%, UI: 0.3566%–0.3723%), and Benin (0.2554%, UI: 0.2496%–0.2611%) experienced the highest burdens. The per capita economic burden estimates were highest in Mauritius (INT$ 37.135, UI: 34.341–39.929), Namibia (INT$ 30.764, UI: 30.099–31.429), and Botswana (INT$ 17.610, UI: 16.187–19.032).

### Estimation and prediction of the economic burden in different WHO regions from 2010 to 2050

**Fig. 1:**
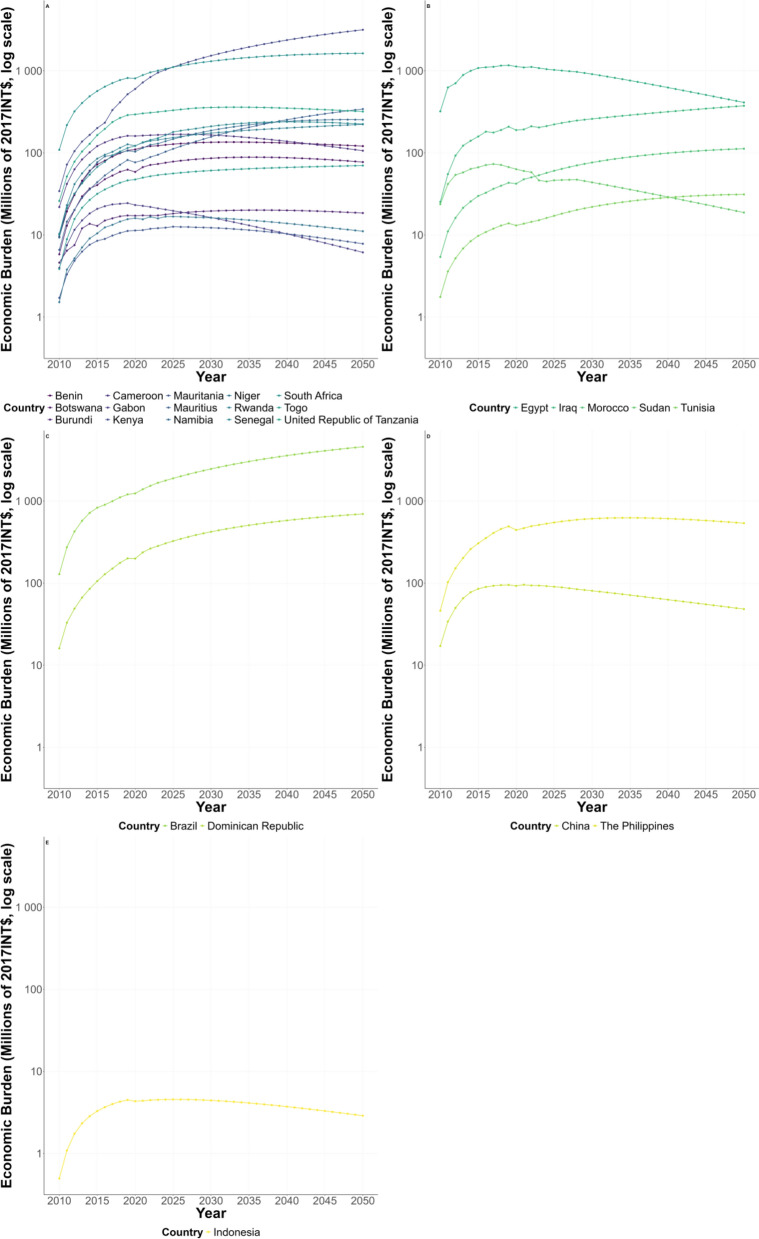
Economic burden in five regions from 2010 to 2050 (**A** African Region; **B** Eastern Mediterranean Region; **C** Region of the Americas; **D** Western Pacific Region; **E** South-East Asia Region)

Estimates and projections of the economic burden in 25 schistosomiasis-endemic countries across five WHO regions from 2010 to 2050 are shown in Fig. 1. In the African region, the economic burden of schistosomiasis was found to be higher in Kenya and South Africa, and lower in other countries. The projections show that the economic burden of Kenya is on the rise, while that of the other countries is relatively stable or decreasing (Fig. 1A). In the Eastern Mediterranean region, the economic burden of schistosomiasis was found to be high in Egypt, but there has been a significant downward trend since 2019. Sudan's economic burden exhibited a declining trend. The economic burden of other countries in the Eastern Mediterranean region was rising, but only slowly (Fig. 1B). In the Americas, the economic burden of schistosomiasis is on the rise, with a significant increase in Brazil (Fig. 1C). In both the Western Pacific and South-East Asia regions, projections showed a downward trend (Fig. 1D, E).

### Estimation and prediction of the human capital loss in the different WHO regions from 2010 to 2050

**Fig. 2:**
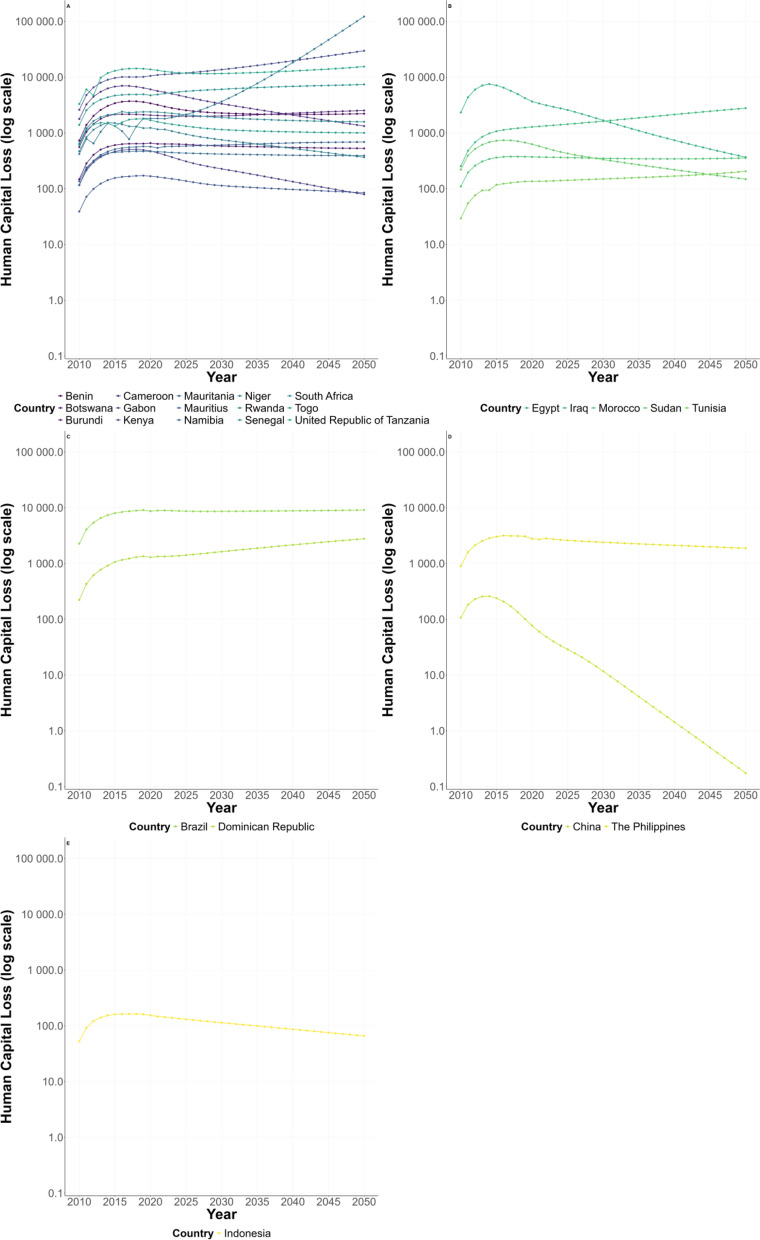
Human capital losses in five regions from 2010 to 2050 (**A** African Region; **B** Eastern Mediterranean Region; **C** Region of the Americas; **D** Western Pacific Region; **E** South-East Asia Region)

Estimates and projections of human capital losses due to schistosomiasis in the 25 countries studied in the five WHO regions are shown in Fig. 2. In the African region, the projections show that human capital losses are gradually declining in most countries, with the exception of Niger, which has seen a significant year-on-year increase (Fig. 2A). In the Eastern Mediterranean region, the human capital loss was gradually declining in Egypt and Sudan, while slowly increasing in other countries (Fig. 2B). In the Americas, the human capital loss was slowly increasing (Fig. 2C), while it was on a downward trend in both the Western Pacific and South-East Asia regions (Fig. 2D, E).

### Sensitivity analysis

**Fig. 3:**
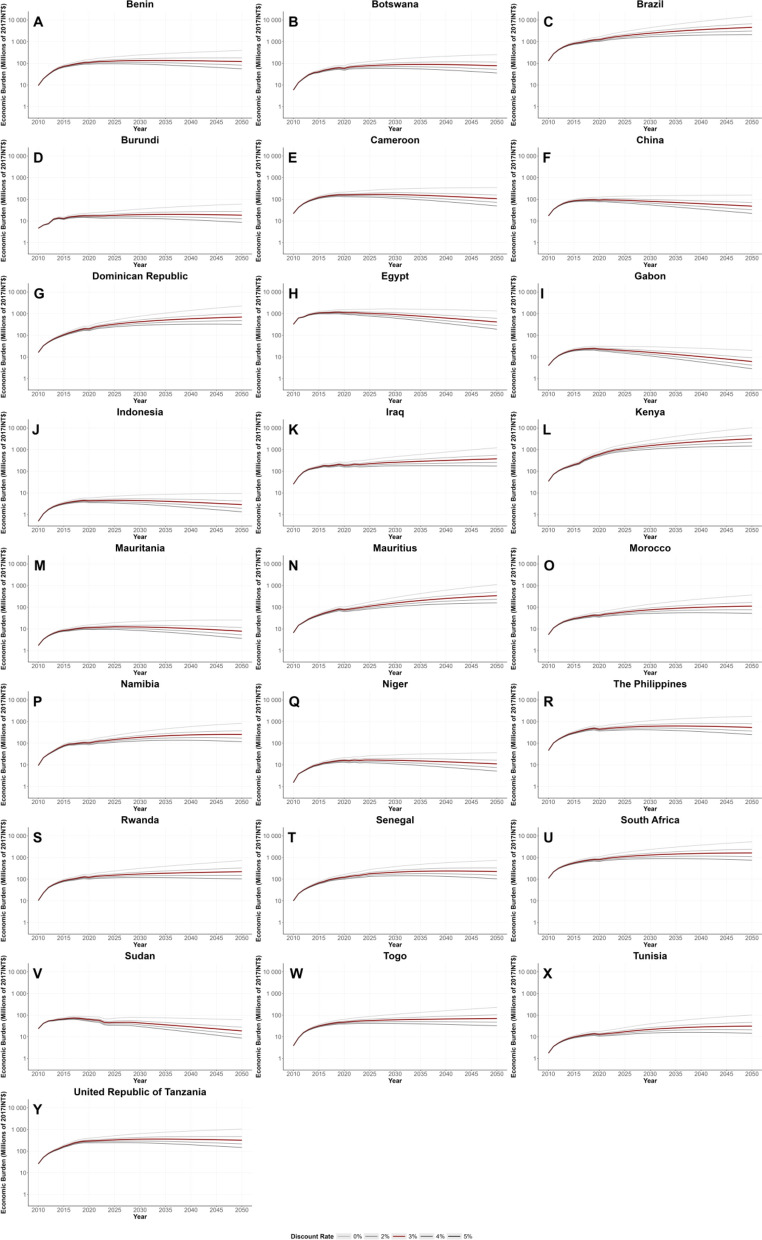
The estimates and projections of the economic burden in 25 countries from 2010 to 2050 under different discount rates, i.e., 0%, 2%, 3%, 4%, and 5% (**A** Benin; **B** Botswana; **C** Brazil; **D** Burundi; **E** Cameroon; **F** China; **G** Dominican Republic; **H** Egypt; **I** Gabon; **J** Indonesia; **K** Iraq; **L** Kenya; **M** Mauritania; **N** Mauritius; **O** Morocco; **P** Namibia; **Q** Niger; **R** The Philippines; **S** Rwanda; **T** Senegal; **U** South Africa; **V** Sudan; **W** Togo; **X** Tunisia; **Y** United Republic of Tanzania)

In order to understand the variations of economic burden contributed by different discount rates, e.g. 0%, 2%, 4%, and 5% , the sensitive analysis was performed. The analysis showed that the estimated total economic burden was INT$ 61,024 million (UI: 60,014–62,033) without discounting; it was INT$ 52,997 million (UI: 52,108–53,886) with a 2% discount rate; it was INT$ 46,310 million (UI: 45,523–47,098) with a 4% discount rate; and it was INT$ 43,388 million (UI: 42,645–44,130) with a 5% discount rate. Estimates for each country and region using discount rates are shown in Table 2. Figure 3 shows the estimations from 2010 to 2021 and projections from 2022 to 2050 of the economic burden for each country under different discount rates.

**Table 2:** Total economic burden of schistosomiasis in 25 countries from 2010 to 2021, using different discount rates

Region	Country	Economic burden discounted at 0% in millions of 2017 INT$ (uncertainty interval)	Economic burden discounted at 2% in millions of 2017 INT$ (uncertainty interval)	Economic burden discounted at 4% in millions of 2017 INT$ (uncertainty interval)	Economic burden discounted at 5% in millions of 2017 INT$ (uncertainty interval)
AFRO	*Regional average*	1604 (751–2862)	1385 (643–2460)	1204 (544–2100)	1124 (489–1977)
	Benin	1046 (1022–1069)	904 (883–924)	785 (768–803)	734 (717–751)
	Botswana	611 (563–659)	529 (487–572)	461 (423–498)	431 (396–466)
	Burundi	191 (188–194)	167 (164–170)	147 (145–150)	138 (136–141)
	Cameroon	1665 (1609–1721)	1447 (1398–1496)	1265 (1222–1309)	1186 (1145–1227)
	Gabon	266 (212–320)	232 (184–280)	204 (162–246)	192 (152–231)
	Kenya	4483 (4454–4513)	3821 (3795–3847)	3276 (3253–3299)	3040 (3019–3062)
	Mauritania	117 (107–128)	102 (93–112)	90 (81–98)	84 (76–92)
	Mauritius	718 (665–771)	619 (573–665)	537 (496–578)	501 (463–539)
	Namibia	1056 (1034–1078)	914 (894–933)	795 (778–813)	743 (727–760)
	Niger	154 (146–162)	133 (126–140)	116 (110–122)	109 (103–115)
	Rwanda	1219 (1214–1224)	1055 (1051–1059)	918 (914–922)	858 (855–862)
	Senegal	1091 (1060–1121)	939 (912–966)	814 (790–838)	759 (737–782)
	South Africa	8320 (8239–8402)	7222 (7150–7293)	6307 (6243–6370)	5907 (5847–5966)
	Togo	459 (455–463)	397 (394–401)	346 (343–349)	323 (320–326)
	United Republic of Tanzania	2668 (2605–2730)	2300 (2245–2354)	1994 (1946–2043)	1861 (1815–1907)
AMRO	*Regional average*	6962 (1810–12,113)	6021 (1556–10,486)	5239 (1345–9133)	4898 (1254–8543)
	Brazil	12,113 (12,038–12,188)	10,486 (10,420–10,552)	9133 (9075–9192)	8543 (8488–8598)
	Dominican Republic	1810 (1740–1880)	1556 (1495–1616)	1345 (1292–1398)	1254 (1204–1303)
EMRO	*Regional Average*	3468 (400–8725)	3047 (402–7722)	2693 (308–6709)	2538 (290–6394)
	Egypt	13,788 (13,578–13,999)	12,127 (11,939–12,315)	10,732 (10,563–10,902)	10,119 (9958–10,280)
	Iraq	2125 (2032–2218)	1853 (1772–1934)	1625 (1554–1696)	1526 (1459–1592)
	Morocco	434 (418–451)	377 (362–391)	329 (316–341)	308 (296–320)
	Sudan	857 (843–871)	758 (745–770)	674 (663–685)	637 (626–647)
	Tunisia	138 (130–146)	120 (113–127)	105 (99–111)	98 (92–104)
SEARO	*Regional average*	45 (37–54)	39 (32–47)	34 (28–41)	32 (26–38)
	Indonesia	45 (37–54)	39 (32–47)	34 (28–41)	32 (26–38)
	*Regional average*	2824 (1084–4564)	2450 (948–3952)	2139 (835–3443)	2002 (785–3220)
WPRO	China	1084 (1081–1086)	948 (946–950)	835 (833–837)	785 (783–787)
	The Philippines	4564 (4543–4584)	3952 (3934–3970)	3443 (3427–3458)	3220 (3205–3235)
Total		61,024 (60,014–62,033)	52,997 (52,108–53,886)	46,310 (45,523–47,098)	43,388 (42,645–44,130)

AFRO: African Regional Office; AMRO: Regional Office for the Americas; EMRO: Eastern Mediterranean Regional Office; SEARO: South-East Asia Regional Office; WPRO: Western Pacific Regional Office

## Discussion

By integrating health factors into a macroeconomic framework, the critical role that health plays in the general economy can be recognized. We are pleased to bring to light several important issues with regard to the economic cost of schistosomiasis that has not previously been reported. The approach presented here should be useful by exposing problem areas and highlighting the potential economic gains from schistosomiasis control [35]. The strengths of this study can be summarized as follows:

- The HAM model has not previously been used to estimate the macroeconomic burden of schistosomiasis;
- This is the first study using a rigorous economic adjustment mechanism that accounts for the impact of schistosomiasis on both human and physical capital accumulation;
- The findings present the varied distribution of both the health and economic burdens of schistosomiasis across countries and regions; and
- Unlike cost-of-illness studies, this study utilizes macroeconomic indicators such as population size, education levels, savings rates and labour force participation—data readily available for international comparisons, enabling a more comparable assessment of the economic burden across countries.

The results presented fill several knowledge gaps and show potential economic gains from schistosomiasis control programme over the past decade and in the future. Although the percentage (0.0174%) of schistosomiasis economic burden relative to GDP across the 25 countries from 2010 to 2021 may seem low, the imposed an economic burden absolute terms (INT$ 49,504 million) is considerable. When comparing the variability of economic losses among regions, it was clear that the economic losses per capita hurt the African region (INT$ 9.652) and the America Region (INT$ 7.739) considerably more than the other parts of the world. The significant differences of the economic loss presented from region to region indicated the international assistance in health in the future need to focus on African region in order to achieve the United Nations (UN) Sustainable Development Goals (SDGs).

In order to further interrupt the vicious cycle between poverty and the diseases in schistosomiasis-endemic regions, we predicted the changes in economic burden and human capital losses due to schistosomiasis in the next 30 years and found a similar pattern between economic burden (Fig. 1) and human capital losses (Fig. 2) from 2022 to 2050. Unfortunately, it is hard to reduce the economic burden or human capital losses in African region compared with other regions. All those findings indicated we still have a long way to go to achieve the SDG1 and SDG3.

Overall, most countries showed a declining trend in human capital losses, reflecting the effectiveness of control measures and a reduction in disease burden. However, notable disparities exist between regions and countries. In the African region, while most countries experience a gradual decline, Niger shows a persistent year-on-year increase, suggesting challenges such as inadequate control measures, worsening environmental conditions, or difficulties in infection control. In the Eastern Mediterranean region, Egypt exhibits a downward trend, whereas other countries face increasing losses, possibly due to variations in disease control efforts, healthcare infrastructure, and socioeconomic factors. The Americas sees a slow increase in the human capital losses, indicating that further improvements in disease control are needed. In contrast, both the Western Pacific and South-East Asia regions demonstrate a steady decline, suggesting that schistosomiasis control strategies in these areas have been relatively effective. Moving forward, it is crucial to consider long-term changes in disease burden in the context of socioeconomic development and climate change, while strengthening targeted interventions in high-risk areas to accelerate global schistosomiasis elimination efforts.

The sensitivity analysis reveals that the estimated total economic burden of schistosomiasis in the 25 endemic countries from 2010 to 2021 varies significantly depending on the applied discount rate. Without discounting, the estimated burden is INT$ 61,024 million, while applying discount rates of 2%, 4%, and 5% progressively reduces the estimates to INT$ 52,997 million, INT$ 46,310 million, and INT$ 43,388 million, respectively. The observed variation underscores the sensitivity of economic burden estimates to discounting assumptions. Since higher discount rates assign lower present value to future costs, the economic impact appears smaller under higher discounting scenarios. However, in the context of schistosomiasis, long-term health effects and productivity losses accumulate over decades, suggesting that lower discount rates might provide a more comprehensive assessment of the true long-term socioeconomic impact. From a public health policy perspective, the choice of discount rate directly influences cost-effectiveness evaluations of schistosomiasis control programs. Lower discount rates (0–2%) emphasize the long-term benefits of investing in elimination programs, as they account for future productivity gains and healthcare cost savings. In contrast, higher discount rates (4–5%) might undervalue long-term consequences, potentially leading to underinvestment in prevention and treatment efforts. Although the aggregated estimates provide a macroeconomic perspective, the discounting effect may vary across countries and regions due to differences in disease burden, healthcare investments, and economic conditions. Countries with higher prevalence and slower control progress might experience more pronounced long-term costs, making the choice of discount rate particularly crucial for policymaking.

Based on our findings regarding the economic burden of schistosomiasis, we recommend that equitable and effective public health investments to reduce this burden are essential for promoting global health and economic well-being. First, increased investment in schistosomiasis control program and innovative strategy to eliminate the disease is needed. This includes research and development of cost-effective technologies, such as vaccines, new drugs, rapid diagnostics, etc. Second, more investment is required in research to identify integrated strategies or combined interventions, including One Health approach [36]. Third, priority countries and regions for schistosomiasis control should be identified based on a joint assessment of the health and economic burdens, balancing health benefits with economic efficiency. Preventive chemotherapy strategies should be implemented in countries with relatively high health and economic burdens, and One Health approach is implemented in the low economic burden regions that are aiming to eliminate schistosomiasis. Equitable and effective public health investments are crucial to reducing the global economic burden of schistosomiasis, accelerating progress towards elimination, and advancing a shared vision for global health [37]. Increased investment in the development of appropriate intervention technologies is critically and urgently needed. Importantly, the schistosomiasis-endemic countries share many common challenges. We call for strengthened collaboration and exchange among these nations to share best practices in schistosomiasis prevention and control.

Our study has several limitations. First, we lacked country-specific data on the cost of schistosomiasis treatment, which had to be extrapolated from Brazilian and Chinese data, assuming a proportional relationship between per capita health expenditure and the treatment cost per schistosomiasis patient, for the accuracy of which we lack evidence. However, while this approach may have led to overestimation or underestimation of treatment costs in specific countries, it is a commonly used method in macroeconomic burden of disease studies. Second, we did not account for changes in labour force participation among family members caring for schistosomiasis patients, or the impact of schistosomiasis on education, thus potentially underestimating the total cost of patient care. Third, we assumed that all schistosomiasis patients require diagnosis and treatment. In fact, access to treatment is limited by socioeconomic and sanitary conditions, potentially leading to an overestimation of treatment costs.

## Conclusions

The global economic burden of schistosomiasis remains substantial without intervention, with considerable variation across countries. Our study highlights the need for increased investment and global collaboration to control schistosomiasis and its associated health and economic burdens. Eliminating schistosomiasis offers significant economic returns, contributing to global economic development. Although this study assumes that the trend of schistosomiasis will continue during the period 2022–2050, we acknowledge that changes may occur in the field. We will continue to monitor disease surveillance data. It is warranted to update and develop the model accordingly in the future.

## Supplementary Information

Supplementary Material 1: Appendix 1. Description of macroeconomic model in the study. Appendix 2. Data description in the study. Table S1. Data gaps across 78 schistosomiasis-endemic countries (in the Appendix 2.10). Table S2. Parameter values and data sources (in the Appendix 2.11). Figure S1. Diagnostic plot of equation (7)：Q-Q plot of residuals (in the Appendix 1.1).

## Data Availability

All data are availed based on requirement from the corresponding author.

## Abbreviations

NTD: Neglected tropical disease
WHO: World Health Organization
LMICs: Low- and middle-income countries
GBD: Global Burden of Disease Study
IHME: Institute for Health Metrics and Evaluation
DALYs: Disability-adjusted life years
HAM: Health-augmented macroeconomic
PPP: Purchasing power parity
GDP: Gross domestic product
YLD: Year of life lost due to disability
YLL: Year of life lost
UI: Uncertainty interval
INT$: International dollars
ILO: International Labour Organization
EPIC: Economic Projections for Illness and Cost of treatment
UN: United Nations
SDGs: Sustainable Development Goals
