# Supplementary material for: Estimation and prediction on the economic burden of schistosomiasis in 25 endemic countries
Source: Infect Dis Poverty. 2025 Jun 16;14:49. doi: 10.1186/s40249-025-01330-8 (PMC12168328; doi:10.1186/s40249-025-01330-8)
Supplement: Supplementary file 1 — Supplementary Material 1: Appendix 1. Description of macroeconomic model in the study. Appendix 2. Data description in the study. Table S1. Data gaps across 78 schistosomiasis-endemic countries (in the Appendix 2.10). Table S2. Parameter values and data sources (in the Appendix 2.11). Figure S1. Diagnostic plot of equation (7)：Q-Q plot of residuals (in the Appendix 1.1). [file 40249_2025_1330_MOESM1_ESM.docx]

**Appendix**

**1 Description of macroeconomic model.**

Detailed model descriptions refer to previous studies. In previous studies, Bloom et al. and Chen et al. applied this model framework to estimate the economic burden of non-communicable diseases in China, Japan, and South Korea; the global economic burden of road traffic injuries; the global economic burden of chronic obstructive pulmonary disease; the global economic burden of cancer; and the economic burden of risk factors such as tobacco and air pollution.

Our study aims to quantify the impact of schistosomiasis on economic output by healthcare expenditures and productivity losses due to mortality and morbidity. For each country, we conducted the following analysis:

Step 1: We identified the disease burden of schistosomiasis (based on mortality, morbidity, and treatment costs).

Step 2: We constructed economic output projections under two scenarios: A status quo scenario representing aggregate output under current conditions with no interventions to reduce schistosomiasis mortality and morbidity; and a counterfactual scenario representing aggregate output if schistosomiasis were completely eliminated at zero cost. Economic projections utilize a macroeconomic production function and can be decomposed into two components: projections of effective labor supply; and projections of physical capital accumulation.

Step 3: We cumulatively summed the difference in annual Gross Domestic Product (GDP) between the two scenarios. The cumulative difference represents the total economic burden:

$$\Delta Y=\sum_{t=2010}^{2050} (\overline{Y}_{t}-Y_{t})(1)$$

**Production function.**

Considering the discrete time evolution (t=1, 2, ...) in the economy, building upon Lucas's work, we considered the following production function for each economy:

$$Y_{t}=A_{t}H_{t}^{1-\alpha}K_{t}^{\alpha} (2)$$

In equation (2), $Y_{t}$ represents aggregate output; $A_{t}$ is the level of technology in year t, which we assume evolves exogenously; $K_{t}$ is the physical capital stock (e.g., machines, factory buildings, etc.); and $H_{t}$ represents total human capital. The parameter $\alpha$ is the elasticity of aggregate output with respect to physical capital. Solow's framework (on which the WHO's original EPIC macroeconomic model was based) considers only physical capital and raw labor as factors of production. However, the aggregate production function recognizes that output is produced not only by these factors but also by effective labor, where health is a crucial determinant.

Physical capital evolves according to the following equation:

$$K_{t+1}=\left( 1-\delta\right)K_{t}+Y_{t}-C_{t}-TC_{t}=\left( 1-\delta\right)K_{t}+s_{t}Y_{t} (3)$$

In equation (3), $\delta$ represents the depreciation rate, $S_{t}$ is the savings rate, ${TC}_{t}$ represents the treatment cost of schistosomiasis, and $C_{t}$ represents the quantity of consumption. From equation (3), the savings rate can be defined as:

$s_{t}=1-\frac{C_{t}+TC_{t}}{Y_{t}}(4)$

Total output $Y_{t}$ is used for three purposes: (i) paying for treatment costs, (ii) consumption, and (iii) savings.

We assumed that the working-age population is defined as those aged 15-64, and this population was further divided into ten 5-year age groups. Thus, we had 20 age-sex groups. Total human capital in the production function can be defined as the sum of the effective labor supply of each age-sex group:

$$H_{t}=\sum_{a} h_{t}^{a}{l_{t}^{a}N}_{t}^{a} (5)$$

In equation (5), $N_{t}^{a}$ represents the number of individuals in age group a, $h_{t}^{a}$ represents the average human capital of individuals in age group a, and $l_{t}^{a}$ represents the labor force participation rate in age group a.

Following the Mincer model, we constructed the average human capital for age group a based on education level and work experience:

$$\ln h_{t}^{a}=\eta_{1}ys_{t}^{a}+\eta_{2}\left( a-ys_{t}^{a}-5 \right)+\eta_{3}\left( a-ys_{t}^{a}-5 \right)^{2} (6)$$

In equation (6), $\eta_{1}$ is the semi-elasticity coefficient of human capital with respect to average years of schooling ${ys}_{t}^{a}$, and $\eta_{2}$ and $\eta_{3}$ are the semi-elasticity coefficients of human capital with respect to experience of the workforce $\left( a-ys_{t}^{a}-5 \right)$ and experience of the workforce squared $\left( a-ys_{t}^{a}-5 \right)^{2}$, respectively. Here, we assumed a school entry age 5 years throughout.

**The impact of schistosomiasis on labor supply**

Referring to Bloom et al. and Chen et al., in the status quo scenario, labor supply can be obtained through $L_{t}^{a}=l_{t}^{a}N_{t}^{a}$.

$$N_{t}^{a}=\left[ 1-\sigma_{t-1}^{a-1} \right]N_{t-1}^{a-1} (7)$$

To validate Equation (7), we computed the predicted value of labor supply over time using Equation (7) and obtained the observed values. We then calculated the differences between them. The mean and standard deviation of the differences were -1.866 and 25.381, respectively. Then we analyzed their distribution using the Shapiro-Wilk normality test and Q-Q plot. The results indicate that the differences follow a normal distribution (W = 0.975, p-value = 0.162), as shown in the Q-Q plot below:


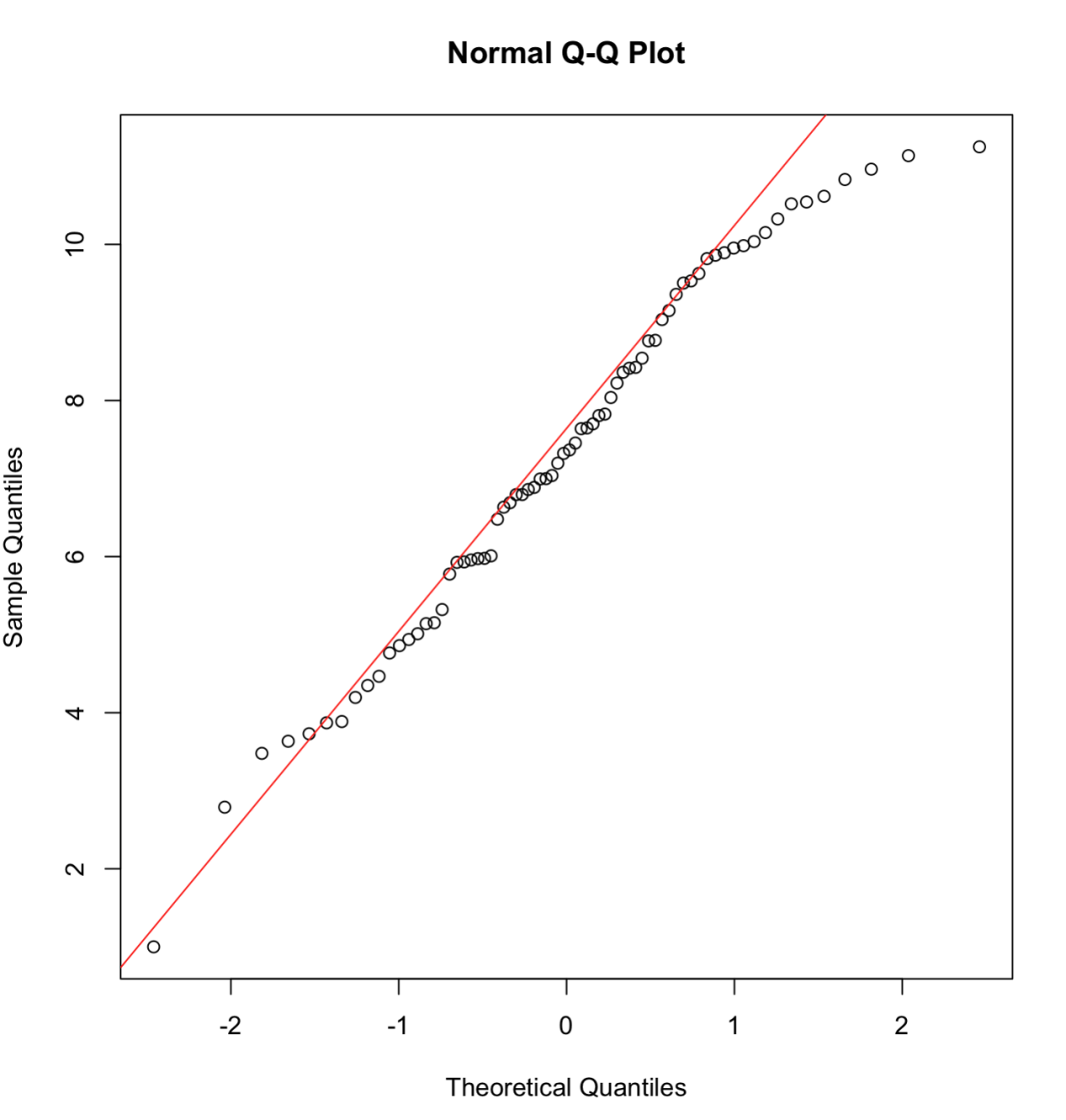


Figure S1: Diagnostic plot of equation (7)：Q-Q plot of residuals

In equation (7), $\sigma_{t}^{a}$ represents the overall mortality rate for age group a in year t. Mortality and morbidity reduce the effective labor supply. The reduction of population size $N_{t}^{a}$ reflects the impact of mortality. $\sigma_{d,t}^{a}$ represents the mortality rate in age group a due to schistosomiasis. $\sigma_{-d,t}^{a}$ represents the overall mortality rate from causes other than schistosomiasis. Then we have:

$$\left( 1-\sigma_{t}^{a} \right)=\left( 1-\sigma_{d,t}^{a} \right)\left( 1-\sigma_{-d,t}^{a} \right)(8)$$

In the counterfactual scenario, variables are represented with an overline. The population size for age group a in year t can be obtained using the following formula:

$$\overline{N}_{t}^{a}=\left[ 1-\sigma_{-d,t-1}^{a-1} \right]\overline{N}_{t-1}^{a-1}(9)$$

$$\overline{N}_{0}^{a}=N_{0}^{a}(10)$$

$$\overline{N}_{t}^{0}=N_{t}^{0}(11)$$

Referencing Bloom et al., the labor loss due to mortality accumulates year by year, as shown in the following formula:

$$\overline{N}_{t}^{a}=\frac{N_{t}^{a}}{\prod_{\tau=0}^{\min\left\{ t,a \right\}-1} \left[ 1-\sigma_{d,t-1-\tau}^{a-1-\tau} \right]}(12)$$

The reduction in labor force participation rate $l_{t}^{a}$ reflects the effects of morbidity, as individuals suffering from illness typically reduce their labor supply, either by reducing working hours or by leaving their jobs. Referencing Bloom et al., the labor force participation rate in the counterfactual scenario $\overline{l}_{t}^{a}$ can be calculated as:

$\overline{l}_{t}^{a}\boldsymbol{\simeq}\frac{l_{t}^{a}}{\prod_{\tau=0}^{\min\left\{ t,a \right\}-1} \left[ 1-p^{\tau}\sigma_{d,t-1-\tau}^{a-1-\tau}\xi^{a-1-\tau} \right]}$(13)

$\xi^{a}$measures the magnitude of the morbidity effect relative to mortality. $p$represents the probability that a schistosomiasis patient cannot recover from the disease and we used the efficacy of the WHO-recommended 40 mg/kg praziquantel treatment regimen to calculate this parameter value^[1]^.

Because the impact of morbidity is difficult to estimate directly, we define:

$\xi^{a}=\frac{loss of labor due to morbidity in age group a}{loss of labor due to mortality in age group a} (14)$

Next, we assumed that for each age group a in any given year, the following equation holds:

$\xi^{a}=\frac{YLD^{a}}{YLL^{a}} (15)$

In equation (15), $YLD^{a}$ represents the years lived with schistosomiasis, and $YLL^{a}$ represents the years of life lost due to schistosomiasis. $\xi^{a}$can be calculated using the corresponding disability-adjusted life year (DALY) data reported in the Global Burden of Disease Study (GBD2021).

**The impact of schistosomiasis on physical capital accumulation.**

Schistosomiasis also hinders physical capital accumulation, as savings are diverted to pay for part of treatment costs. According to Bloom et al. and Chen et al., physical capital accumulation in the counterfactual can be written as:

$$\bar{K}_{t+1}=\bar{s}_{t}\bar{Y}_{t}+\left( 1-\delta\right)\bar{K}_{t} (16)$$

$$\bar{s}_{t}\bar{Y}_{t}=s_{t}\bar{Y}_{t}+\chi TC_{t} (17)$$

For more details, please refer to Bloom et al. and Chen et al.

To solve this model, we first used the predicted GDP, effective labor supply, and physical capital stock in the status quo scenario (2010-2050) to calibrate the technological level parameter $A_{t}$. Then, we simulated the effective labor supply and physical capital stock in the counterfactual scenario, combined with the estimated technological level, and finally calculated the counterfactual GDP.

1. **Data description.**

This section describes the methodology used to estimate health and economic variables in the status quo scenario (2010-2050).

**Education**

Educational attainment data for specific sex-age groups were obtained from the Barro-Lee Educational Attainment Database, which provides educational attainment data in five-year age groups up to 2010. While data for specific sex-age groups are unavailable for the years 2010-2050, the database offers projections for the 15-64 age group from 2015-2040. We estimated values for specific age groups by assuming that educational attainment grew at the same rate for each age group. Because the Barro-Lee Educational Attainment Database provides data in five-year intervals, linear interpolation was adopted to expand the estimates for each year.

**Mortality and morbidity**

Mortality, morbidity and DALYs (YLLs and YLDs) data due to schistosomiasis were obtained from the recently updated Global Burden of Disease (GBD 2021). To extend the estimates beyond 2021, we assume that the schistosomiasis mortality rate in each country grows at the same rate observed from 2010 to 2021. The estimation of morbidity rates and DALYs followed the same methodology.

**GDP**

GDP estimates (in constant 2017 international dollars) for 2010-2022 were obtained from the World Bank database. The GDP growth rates from 2023 to 2028 are sourced from the International Monetary Fund (IMF). We assume that the GDP growth rate beyond 2028 will remain the same as in 2023-2028.

**Physical capital**

The physical capital stock (in 2017 international dollars) for each country was obtained from the Penn World Table database. The elasticity of aggregate output with respect to capital stock was also sourced from the Penn World Table database and this elasticity is equal to the share of labor compensation in GDP.

**Population.**

Population data for specific sex-age groups from 2010 to 2050 were obtained from the World Population Prospects 2022. The following age groups were considered in the simulation: 15-19, 20-24, 25-29, 30-34, 35-39, 40-44, 45-49, 50-54, 55-59, and 60-64.

**Labor participation**

Labor force participation rates (by five-year age groups) for each country from 2010 to 2022 were obtained from the International Labour Organization (ILO) database. Data for some schistosomiasis-endemic countries were partially missing. Linear and nearest-neighbor interpolation methods were used to impute the missing values. We assume that the growth rate of labor force participation beyond 2022 will remain consistent with that observed from 2010 to 2022.

**Saving rate and health expenditure**

We obtained data on savings rates from 2010 to 2022 and per capita health expenditure from 2010 to 2020 from the World Bank database. For some schistosomiasis-endemic countries, the savings rate data were partially missing. We used linear interpolation and nearest-neighbor interpolation methods to fill in the missing data. To project beyond 2022, we assumed that the savings rate would remain constant (based on the average from 2010 to 2022). For per capita health expenditure, we assumed it would grow at the average rate observed from 2010 to 2020.

**Treatment costs**

Total treatment costs of schistosomiasis in Brazil were based on Nascimento et al., which included 26,499 schistosomiasis carriers, 397 patients with hepatosplenomegaly, 48 with neurological disorders, and 284 hospitalized patients in 2015. Total treatment costs included US$ 2.1 million for diagnosis of schistosomiasis and its main complications, and US$ 147,513.24 for treatment of hepatosplenic and neurological infections. The treatment costs of schistosomiasis in China were derived from Li Yiting et al. We can calculate the per case treatment cost for patients with Schistosomasis mansoni and Schistosomasis japonicum separately. We assumed that per case treatment cost in all countries was proportional to per capita health expenditure. Then we calculated the per case treatment cost for the countries with data and extrapolated costs for countries without data. We utilized China's proportion to estimate treatment costs for countries endemic with schistosomiasis japonica, while applying Brazil's proportion to estimate treatment costs for countries endemic with other forms of schistosomiasis. The total treatment costs of schistosomiasis were extrapolated based on population size and prevalence, using the same methodology as previous studies.

$$Per case treatment cost_{d}=\beta_{d}*Per capita health expenditures (18)$$

In Equation (18), $\beta_{d}$ represents the proportion of per case treatment cost to per capita health expenditure, assumed to be constant across all countries. The total treatment costs can be calculated as:

$$Treatment costs_{d}=\beta_{d}*prevalence_{d}*per capita health expenditures*population (19)$$

Equation (19) calculates the per capita treatment cost of schistosomiasis treatment by multiplying the treatment cost per case by the prevalence of schistosomiasis. To ensure comparability of estimates across countries, all treatment costs were converted to the base year of 2017.

**Discount rate.**

The 3% discount rate is somewhat standard in global health and recommended by the Panel on Cost-Effectiveness in Health and Medicine. However, the appropriate discount rate depends on the current economic environment and varies across countries. For example, the discount rate for low- and middle-income countries should be larger than for high-income countries, such as 5%. Guidelines for economic evaluation from 17 out of 22 countries recommend discount rates ranging from 1.5% to 5% for country-specific discount rates. Therefore, we chose 3% as the discount rate in our main analysis, and provide economic burden projections discounted at 0%, 2%, 4%, and 5%.

**Data Gaps**

Table S1 presents the data availability across 78 schistosomiasis-endemic countries.

Table S1: Data gaps across 78 schistosomiasis-endemic countries.

| **Country** | **DALY** | **Prevalence** | **GDP** | **Treatment cost** | **Education** | **Physical capital** | **Population** | **Saving rate** | **Labour participation rate** | **Health expenditure per capita** | **α** | **Consumption** |
| --- | --- | --- | --- | --- | --- | --- | --- | --- | --- | --- | --- | --- |
| **Algeria** |  |  |  |  |  |  |  |  |  |  | **×** |  |
| **Angola** |  |  |  |  | **×** |  |  |  |  |  |  |  |
| **Antigua and Barbuda** |  |  |  |  | **×** |  |  |  | **×** |  |  |  |
| **Benin** |  |  |  |  |  |  |  |  |  |  |  |  |
| **Botswana** |  |  |  |  |  |  |  |  |  |  |  |  |
| **Brazil** |  |  |  |  |  |  |  |  |  |  |  |  |
| **Burkina Faso** |  |  |  |  | **×** |  |  |  |  |  |  |  |
| **Burundi** |  |  |  |  |  |  |  |  |  |  |  |  |
| **Cambodia** |  |  |  |  |  |  |  |  |  |  | **×** |  |
| **Cameroon** |  |  |  |  |  |  |  |  |  |  |  |  |
| **Central African Republic** |  |  |  |  |  |  |  | **×** | **×** |  |  |  |
| **Chad** |  |  |  |  | **×** |  |  |  |  |  |  |  |
| **China** |  |  |  |  |  |  |  |  |  |  |  |  |
| **Congo** |  |  |  |  |  |  |  |  |  |  | **×** |  |
| **Côte d'Ivoire** |  |  |  |  |  |  |  |  |  |  |  |  |
| **Democratic Republic of the Congo** |  |  |  |  |  |  |  |  |  |  | **×** |  |
| **Djibouti** |  |  |  |  | **×** |  |  |  |  |  |  |  |
| **Dominican Republic** |  |  |  |  |  |  |  |  |  |  |  |  |
| **Egypt** |  |  |  |  |  |  |  |  |  |  |  |  |
| **Equatorial Guinea** |  |  |  |  | **×** |  |  |  | **×** |  | **×** |  |
| **Eritrea** |  |  |  |  | **×** |  |  | **×** | **×** |  |  |  |
| **Eswatini** |  |  |  |  | **×** |  |  |  |  |  |  |  |
| **Ethiopia** |  |  |  |  | **×** |  |  |  |  |  |  |  |
| **Gabon** |  |  |  |  |  |  |  |  |  |  |  |  |
| **Gambia** |  |  |  |  |  |  |  |  |  |  | **×** |  |
| **Ghana** |  |  |  |  |  |  |  |  |  |  | **×** |  |
| **Guinea** |  |  |  |  | **×** |  |  |  |  |  |  |  |
| **Guinea-Bissau** |  |  |  |  | **×** |  |  |  |  |  | **×** |  |
| **Indonesia** |  |  |  |  |  |  |  |  |  |  |  |  |
| **Iran (Islamic Republic of)** | **×** |  |  |  |  |  |  | **×** |  |  |  |  |
| **Iraq** |  |  |  |  |  |  |  |  |  |  |  |  |
| **Jordan** |  |  |  |  |  |  |  |  |  |  |  | **×** |
| **Kenya** |  |  |  |  |  |  |  |  |  |  |  |  |
| **Lao People's Democratic Republic** |  |  |  |  |  |  |  |  |  |  |  | **×** |
| **Lebanon** |  |  |  |  | **×** |  |  |  |  |  |  |  |
| **Liberia** |  |  |  |  |  |  |  | **×** |  |  | **×** | **×** |
| **Libya** |  |  |  | **×** |  | **×** |  |  | **×** | **×** | **×** |  |
| **Madagascar** |  |  |  |  | **×** |  |  |  |  |  |  |  |
| **Malawi** |  |  |  |  |  |  |  |  |  |  | **×** |  |
| **Mali** |  |  |  |  |  |  |  |  |  |  | **×** |  |
| **Mauritania** |  |  |  |  |  |  |  |  |  |  |  |  |
| **Mauritius** |  |  |  |  |  |  |  |  |  |  |  |  |
| **Morocco** |  |  |  |  |  |  |  |  |  |  |  |  |
| **Mozambique** |  |  |  |  |  |  |  | **×** |  |  |  |  |
| **Namibia** |  |  |  |  |  |  |  |  |  |  |  |  |
| **Niger** |  |  |  |  |  |  |  |  |  |  |  |  |
| **Nigeria** |  |  |  |  | **×** |  |  |  |  |  |  |  |
| **Oman** |  |  |  |  | **×** |  |  |  |  |  |  |  |
| **Philippines** |  |  |  |  |  |  |  |  |  |  |  |  |
| **Rwanda** |  |  |  |  |  |  |  |  |  |  |  |  |
| **Saint Lucia** |  | **×** |  | **×** |  |  |  |  |  |  |  |  |
| **Sao Tome and Principe** |  |  |  |  | **×** |  |  |  |  |  |  |  |
| **Saudi Arabia** |  |  |  | **×** |  |  |  |  |  | **×** |  |  |
| **Senegal** |  |  |  |  |  |  |  |  |  |  |  |  |
| **Sierra Leone** |  |  |  |  |  |  |  |  |  |  |  |  |
| **Somalia** |  |  |  |  | **×** |  |  |  |  |  | **×** |  |
| **South Africa** |  |  |  |  |  |  |  |  |  |  |  |  |
| **South Sudan** |  |  |  |  | **×** |  |  |  |  |  | **×** |  |
| **Sudan** |  |  |  |  |  |  |  |  |  |  |  |  |
| **Suriname** |  |  |  |  | **×** |  |  | **×** |  |  |  |  |
| **Syrian Arab Republic** |  |  |  |  | **×** |  |  | **×** |  |  | **×** |  |
| **Togo** |  |  |  |  |  |  |  |  |  |  |  |  |
| **Tunisia** |  |  |  |  |  |  |  |  |  |  |  |  |
| **Türkiye** |  | **×** |  | **×** |  |  |  |  |  |  |  |  |
| **Uganda** |  |  |  |  |  |  |  |  |  |  | **×** |  |
| **United Republic of Tanzania** |  |  |  |  |  |  |  |  |  |  |  |  |
| **Venezuela (Bolivarian Republic of)** |  |  |  |  |  |  |  |  |  |  |  | **×** |
| **Yemen** |  |  |  | **×** |  |  |  |  |  | **×** | **×** | **×** |
| **Zambia** |  |  |  |  |  |  |  |  |  |  |  | **×** |
| **Zimbabwe** |  |  |  | **×** |  |  |  |  |  | **×** |  |  |
| **Guadeloupe** | **×** |  |  |  |  |  |  |  |  |  |  |  |
| **India** | **×** |  |  |  |  |  |  |  |  |  |  |  |
| **Japan** | **×** |  |  |  |  |  |  |  |  |  |  |  |
| **Malaysia** | **×** |  |  |  |  |  |  |  |  |  |  |  |
| **Martinique** | **×** |  |  |  |  |  |  |  |  |  |  |  |
| **Montserrat** | **×** |  |  |  |  |  |  |  |  |  |  |  |
| **Puerto Rico** | **×** |  |  |  |  |  |  |  |  |  |  |  |
| **Thailand** | **×** |  |  |  |  |  |  |  |  |  |  |  |

Note: The savings rates of Côte d'Ivoire and Sierra Leone were negative in certain years, rendering them incompatible with the model. Consequently, these two countries were excluded from the analysis. Parameter $\alpha$ is elasticity of aggregate output with respect to capital stock. The × symbol indicates missing data for the respective country.

**Other parameter values and data sources.**

Table S2 shows the parameter values and data sources used in the model. The parameter definitions are consistent with Bloom et al. and Chen et al.

Table S2: Parameter values and data sources.

| **Parameter** | **Definition** | **Value** | **Source** |
| --- | --- | --- | --- |
| $\alpha$ | Capital share | Country specific | Penn World Table (2021) |
| $\delta$ | Depreciation rate | 0.05 | Grossmann et al. (2013) |
| $\eta_{1}$ | Mincer elasticity of education | 0.091 | Psacharopoulos and Patrinos (2018) |
| $\eta_{2}$ | First-degree Mincer elasticity of experience | 0.1301 | Heckman et al. (2006) |
| $\eta_{3}$ | Second-degree Mincer elasticity of experience | -0.0023 | Heckman et al. (2006) |
| $\chi$ | Fraction of treatment cost financed out of saving | Set as saving rate | World Bank (2024) |

**Currency unit conversion.**

We converted all economic variables to constant 2017 international dollars using market exchange rates, purchasing power parity (PPP) exchange rates, and inflation rates from the World Bank and the International Monetary Fund.

**References**

1. Zwang J, Olliaro PL. Clinical Efficacy and Tolerability of Praziquantel for Intestinal and Urinary Schistosomiasis—A Meta-analysis of Comparative and Non-comparative Clinical Trials. PLoS Negl Trop Dis 2014;8(11):e3286.
